# Ambient temperature and dengue hospitalization in Brazil: A 10-year period case time series analysis

**DOI:** 10.1097/EE9.0000000000000360

**Published:** 2024-12-30

**Authors:** Rafael Lopes, Xavier Basagaña, Leonardo S. L. Bastos, Fernando A. Bozza, Otavio T. Ranzani

**Affiliations:** a Instituto de Física Teórica - IFT, UNESP, São Paulo, Brazil; b Barcelona Institute for Global Health, ISGlobal, Universitat Pompeu Fabra, CIBER Epidemiología y Salud Pública, Barcelona, Spain; c Department of Industrial Engineering (DEI), Pontifical Catholic University of Rio de Janeiro (PUC-Rio), Rio de Janeiro, State of Rio de Janeiro, Brazil; d National Institute of Infectious Disease Evandro Chagas (INI), Oswaldo Cruz Foundation (FIOCRUZ), Rio de Janeiro, State of Rio de Janeiro, Brazil; e Pulmonary Division, Heart Institute (InCor), HCFMUSP, Faculdade de Medicina da Universidade de São Paulo, São Paulo, Brazil

**Keywords:** Dengue, Heat, Hospitalization, Temperature

## Abstract

**Background:**

Dengue has an increased worldwide epidemic potential with the global rising temperature due to climate change. Heat and rainfall are known to influence seasonal patterns of dengue transmission over the course of weeks to months. However, there is a gap in knowledge about the short-term effect of heat on dengue severity. We aimed to quantify the effect of ambient temperature on dengue hospitalization risk in Brazil.

**Methods:**

Daily dengue hospitalization counts and average daily ambient temperature from 2010 to 2019 were analyzed from Brazil. We applied the case time series design combined with a distributed lag nonlinear model framework to estimate relative risk (RR) estimates for dose–response and lag–response structures for the association of temperature and dengue hospitalization. We estimate the overall dengue hospitalization RR for the whole country as well as for each of the five macroregions.

**Results:**

A total of 579,703 hospital admissions due to dengue occurred between 2010 and 2019. We observed a positive association between high temperatures and a high risk of hospitalization across the country. Under extreme heat (95th percentile of temperature), the RR was 3.47 (95% confidence interval: 2.88, 4.19) compared with minimum hospitalization risk. This association was mainly driven by an immediate effect of heat (lag 0) and was similar for the Northeast, Center-West, Southeast, and South regions, but unclear for the North. The risk was of greater magnitude among females and those aged ≥65 years.

**Conclusion:**

Short-term high temperatures are associated with an increase in the risk of hospitalization by dengue.

**What this study adds**

This study provides the first nationwide evidence of a short-term association between high ambient temperatures and increased risk of dengue-related hospitalizations in Brazil. Applying a case time series design and distributed lag nonlinear models, we found that extreme heat significantly raises the risk of hospitalization due to dengue, particularly within the first 2 days following exposure. Our findings highlight the immediate impact of high temperatures on dengue severity, which exacerbates health system burdens, complementing the well-known long-term effects of temperature on dengue transmission.

## Introduction

Dengue fever is a major global public health threat, present largely in the tropics, with an estimated burden over 390 million infections annually.^1,2^ Brazil is among the most impacted countries, with over 25 million reported cases following the implementation of the surveillance system in Brazil.^3,4^ Additionally, the costs associated with this burden are high, estimated at USD 159 million for the treatment and assistance to dengue cases, and USD 10 million on severe dengue between 2000 and 2015.^3^ The impressive burden of dengue in Brazil is also observed in several other low-income and middle-income countries.^5^

Dengue incidence is influenced by temperature and rainfall, which affect the mosquito life cycle, contributing to an increased transmission risk during warmer and wetter periods.^1,4–9^ A systematic review and meta-analysis published in 2023, which pooled 106 studies,^10^ found sufficient evidence to show increased relative risk (RR) in dengue incidence for high temperatures (pooled –RR: 1.13; 95% confidence interval (CI): 1.11, 1.16, for 1 °C increase), though the evidence for heatwave events was limited (pooled RR: 1.08; 95% CI: 0.95, 1.23). Most studies used a monthly time resolution for the exposure, usually exploring lag effects of up to 1 year. The magnitude of the association reported seemed to depend on the climate zone, with a higher incidence risk observed in tropical monsoon and humid subtropical climate zones.^10^ Projections suggest that dengue incidence, along with other mosquito-borne (mainly *Aedes*-born) diseases such as Chikungunya and Zika, could see increased epidemic potential due to global temperature rises by climate change.^7,11^

Dengue fever can range in severity. Approximately one-quarter of those infected manifest as clinical or subclinical disease,^1^ but sometimes requiring hospital admission and vital organ support.^12^ The primary mechanisms underlying severe dengue include dehydration and coagulation disorders,^12–14^ both of which can be aggravated by heat.^15,16^ Although the literature is clear about the association between temperature and dengue incidence, there is no evidence for the association between ambient temperature and severe dengue. Based on the reported association of short-term extremes of temperature and all-cause hospitalizations,^17–19^ we hypothesized that short-term high ambient temperatures are positively associated with the risk of hospitalization due to dengue. We aimed to evaluate the association between ambient temperature and dengue hospitalizations in Brazil from 2010 to 2019.

## Material and methods

### Study design

We conducted a case time series analysis to evaluate the association between ambient temperature and dengue hospitalizations in Brazil. A complete description of the model used is given in the Supplemental Material; http://links.lww.com/EE/A318.

### Study area

Brazil, located in South America, has a population of approximately 211 million spread over an area of 8.5 million km². The country is divided into 26 states and one federal district.^20^ These 27 states are grouped in five administrative macroregions: North (seven states), Northeast (nine states), Center-West (four states), Southeast (four states), and South (three states). Brazil lies predominantly within the tropics and exhibits three main Köppen climate types, tropical, dry or semi-arid, and humid subtropical, and 12 climate subtypes.^21^

### Outcome and covariates

The primary outcome of this study is dengue hospitalization. Data were obtained from the Brazilian hospital admission system (Sistema de Informações Hospitalares), a nationwide database that includes individual-level data for all hospitalizations covered by the universal healthcare system (Sistema Único de Saúde) in Brazil. We defined a dengue hospitalization by the following International Classification of Diseases-10 codes: A90, A91, A97, A970, A971, A972, ‘A979 (definitions provided in Supplementary Material; http://links.lww.com/EE/A318). We constructed a daily time series by aggregating the number of events by date of hospitalization for each municipality of residence.

We used the total number of dengue cases as a covariate in a sensitivity analysis. Data were retrieved from the national dengue surveillance system (Sistema de Informação de Agravos de Notificação-Dengue), which records notifications for suspected or confirmed dengue cases. We selected confirmed cases and built a daily time series by aggregating the number of cases by date of symptom onset at each municipality. To incorporate this covariate into the model, we derived a 7-day moving average of the dengue cases series, an estimated time of progression from symptoms onset to hospitalization.

Both databases are publicly accessible and comply with ethical principles regarding open data. According to Brazilian Ethics Resolution No. 510/2016, the use of this data did not require ethical approval.

### Temperature exposure assessment

Our exposure of interest is the daily average temperature in each Brazilian municipality. We derived this using the hourly 2-m temperature data (gridded 0.1º × 0.1º) from ERA5-Land reanalysis products, freely available through the Copernicus Climate Data Store.^22^ The daily mean temperature for each municipality was calculated by averaging the temperature of each grid cell and weighting it according to the municipality’s area, using the “exactextractr” R package.^23^

To validate the temperature estimates from ERA5-Land for Brazil, we compared them with data from monitoring stations at the municipal level. We used data from 389 stations (269 automatic and 120 manual) provided by the National Institute of Meteorology,^24^ selecting only stations with at least 90% of days with complete data during the study period. This analysis across 340 municipalities covering all 27 states and 10-year period showed a Pearson correlation coefficient of 0.94, *R*^2^ of 0.90, and root mean square deviation of 1.54 °C. Further information is available in Supplemental Material; http://links.lww.com/EE/A318.

### Data analysis

We estimated the association between ambient temperature and dengue hospitalizations at the national level, within each macroregion, and across the 27 states using a case time series analysis with conditional quasi-Poisson regression and distributed lag nonlinear models (DLNMs) based on municipality-specific temperature–dengue hospitalization series.^25–27^ The case time series design allows the estimation of exposure–response functions within each region (i.e., Brazil, macroregions, and states), while utilizing high-resolution data at the municipality level. Another advantage of this approach is its inherent control for time-invariant confounding factors, such as sex and chronic comorbidities.^27^ We modeled the temperature–dengue hospitalization association using the DLNM framework, allowing for nonlinear exposure–response relationships and lagged effects. The exposure–response association was modeled with natural splines using two knots equally spaced along the dose–response curve. The lag–response curve was evaluated considering 7 lag days and modeled with two knots equally spaced on the logarithmic scale. We selected the cross-basis parameters for the dose–response curve based on the literature and validated them using quasi-Akaike’s criterion (q-AIC, Table A1; http://links.lww.com/EE/A318), selecting the lowest q-AIC for the main analysis. We selected the cross-basis parameters for the lag–response curve based on what the literature has been using for the 7-day lag time period.^28,29^ To control for temporal trends, we used the matching strata from the case time series design defined by municipality, month, and day of the week, and further adjusted for the long-term trends using natural splines with 7 degrees of freedom per year over the study period.^17,30^ We also evaluated the association for the hospitalizations by sex and age (<65 and ≥65 years) creating a times series for each subgroup.

### Sensitivity analyses

We conducted three sensitivity analyses to explore the DLNM parametrization and account for the number of dengue cases at risk for hospitalization. In the first sensitivity analysis, we parameterized the cross-basis with three knots equally spaced along the dose–response curve, the second lowest q-AIC, while maintaining two knots equally spaced on the logarithmic scale for the lag–response structure. In the second sensitivity analysis, we used two knots equally spaced on the dose curve (as in the main analysis) but applied three knots equally spaced on the logarithmic scale for the lag–response structure. This choice was to allow more flexibility in the lag structure than the prespecified because we have no other pattern for comparison in the literature evaluating the outcome dengue hospitalization. In the third sensitivity analysis, we included in the main models the 7-day moving average of the confirmed dengue cases, to account for the number of dengue cases in the city and to reinforce that the effect of temperature on dengue incidence is different for its effect on hospitalization.

We report the overall cumulative RR compared with the minimum hospitalization temperature (MHT), defined as the temperature at which the temperature–hospitalization association is minimum. We also report RR for lag effects up to 7 days at the 50th and 95th percentiles of the temperature distribution of each region of interest, centered on the MHT.

All the analyses were conducted using R Software, version 4.3.3.

## Results

### Climate data description

**Figure 1.**
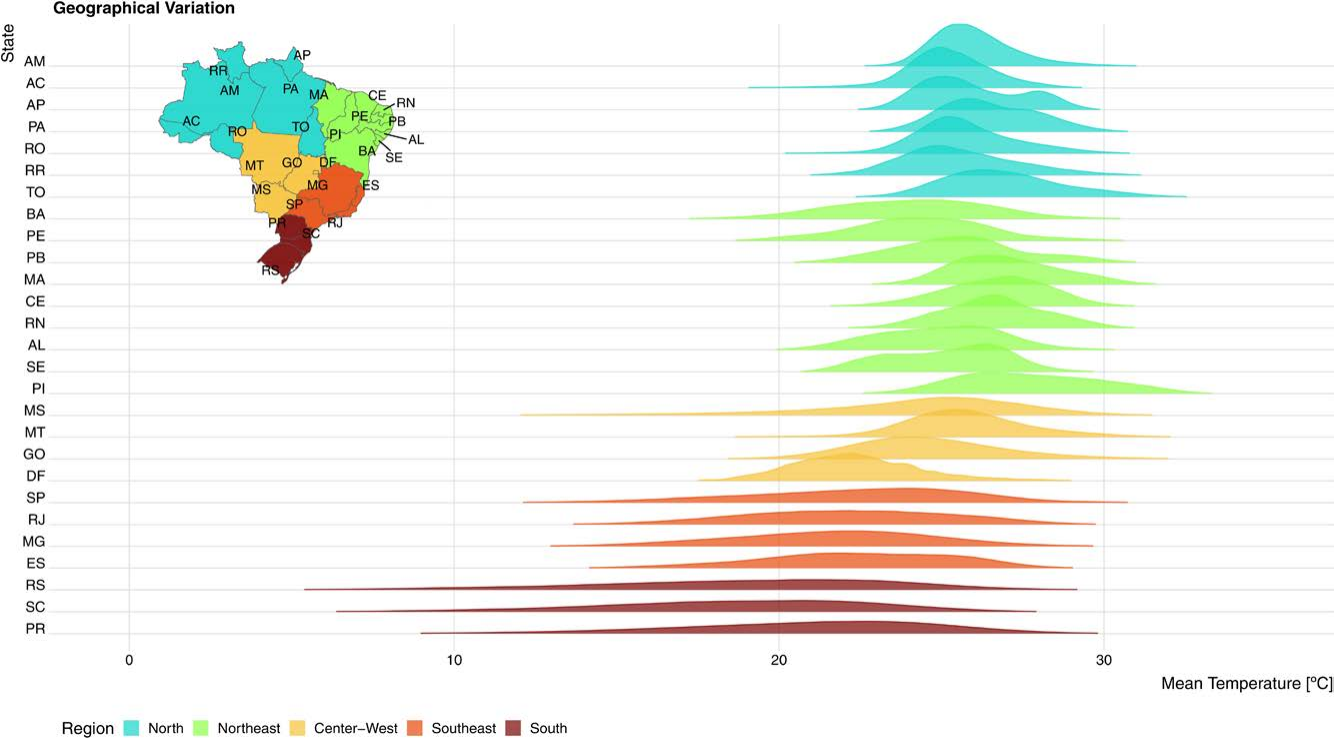
Mean temperature density distribution over each state. On the *y* axis is each of the state abbreviations and on the *x* axis is the range of mean temperature in degree Celsius. The height of each curve is the cumulative density of the mean temperature at that specific degree. The colors are given by each macro administrative region. The inset map gives the location of each of the state codes in Brazilian geography.

A descriptive summary of the mean daily temperature for each state, macroregion, and the entire country over the 10-year period is provided in Table A2; http://links.lww.com/EE/A318. During this period, the temperature ranged from −0.09 to 34.8 ºC. Figure 1 illustrates the distribution of daily mean temperature across all states.

### Dengue hospitalization data description

Between 2010 and 2019, a total of 11,290,103 confirmed dengue cases were reported in the national surveillance system. Our analysis included 579,703 dengue-related hospital admissions from 5565 municipalities, yielding a hospitalization rate of 5.13%.

Table 1 presents a summary of the hospitalization data for each macroregion. The overall mean age of patients hospitalized for dengue was 30 years. We observed regional differences with the mean age of hospitalization ranging from 25 years in the Northeast region to 37 years in the South region, a difference of 12 years. The sex ratio and other demographic differences across regions align with the national demographic statistics, as shown in Table A3; http://links.lww.com/EE/A318. The overall in-hospital mortality was low (0.6%). Figure A1; http://links.lww.com/EE/A318 provides a visual representation of the time series for dengue hospitalizations across different regions.

**Table 1.** Characteristics description of dengue hospitalization data

Variables	n	Brazil (n = 579,703)	North (n = 75,304)	Northeast (n = 224,085)	Center-West (n = 100,056)	Southeast (n = 155,405)	South (n = 24,853)
Age (years); mean (IQR)	579,703	30 (15–49)	27 (15–44)	25 (12–45)	36 (20–53)	34 (17–54)	37 (21–55)
Age categories (years); n (%)	579,703						
0–1		8,752 (1.5%)	1,309 (1.7%)	4,457 (2.0%)	1,058 (1.1%)	1,726 (1.1%)	202 (0.8%)
1–9		73,507 (13%)	9,401 (12%)	37,354 (17%)	8,438 (8.4%)	16,620 (11%)	1,694 (6.8%)
10–17		91,786 (16%)	12,562 (17%)	41,991 (19%)	12,155 (12%)	22,171 (14%)	2,907 (12%)
18–39		192,819 (33%)	29,153 (39%)	72,769 (32%)	34,330 (34%)	48,009 (31%)	8,558 (34%)
40–59		126,059 (22%)	14,863 (20%)	39,640 (18%)	26,825 (27%)	38,268 (25%)	6,463 (26%)
60–79		71,395 (12%)	6,737 (8.9%)	22,314 (10.0%)	14,656 (15%)	23,494 (15%)	4,194 (17%)
80+		15,385 (2.7%)	1,279 (1.7%)	5,560 (2.5%)	2,594 (2.6%)	5,117 (3.3%)	835 (3.4%)
Self-reported race, n (%)	396,624						
Black		12,467 (3.1%)	1,042 (2.3%)	3,946 (2.7%)	1,187 (1.9%)	5,760 (4.9%)	532 (2.6%)
Pardo		244,869 (62%)	39,925 (87%)	120,476 (81%)	34,973 (55%)	45,113 (38%)	4,382 (21%)
Indigenous		1,092 (0.3%)	306 (0.7%)	116 (<0.1%)	595 (0.9%)	62 (<0.1%)	13 (<0.1%)
White		129,031 (33%)	3,893 (8.5%)	19,700 (13%)	24,396 (39%)	65,746 (55%)	15,296 (74%)
Asian		9,165 (2.3%)	700 (1.5%)	4,003 (2.7%)	2,116 (3.3%)	1,991 (1.7%)	355 (1.7%)
Missing		183,079	29,438	75,844	36,789	36,733	4,275
Sex, n (%)	579,703						
Female		310,308 (54%)	38,225 (51%)	121,175 (54%)	54,916 (55%)	82,550 (53%)	13,442 (54%)
Male		269,395 (46%)	37,079 (49%)	102,910 (46%)	45,140 (45%)	72,855 (47%)	11,411 (46%)
Duration of hospitalisation (days); median (IQR)	579,703	3 (2–4)	2 (2–3)	3 (2–4)	2 (2–3)	3 (2–4)	2 (2–3)
In-hospital mortality, n (%)	579,703	3,436 (0.6%)	281 (0.4%)	958 (0.4%)	548 (0.5%)	1,510 (1.0%)	139 (0.6%)

### Temperature–dengue hospitalization association

**Figure 2.**
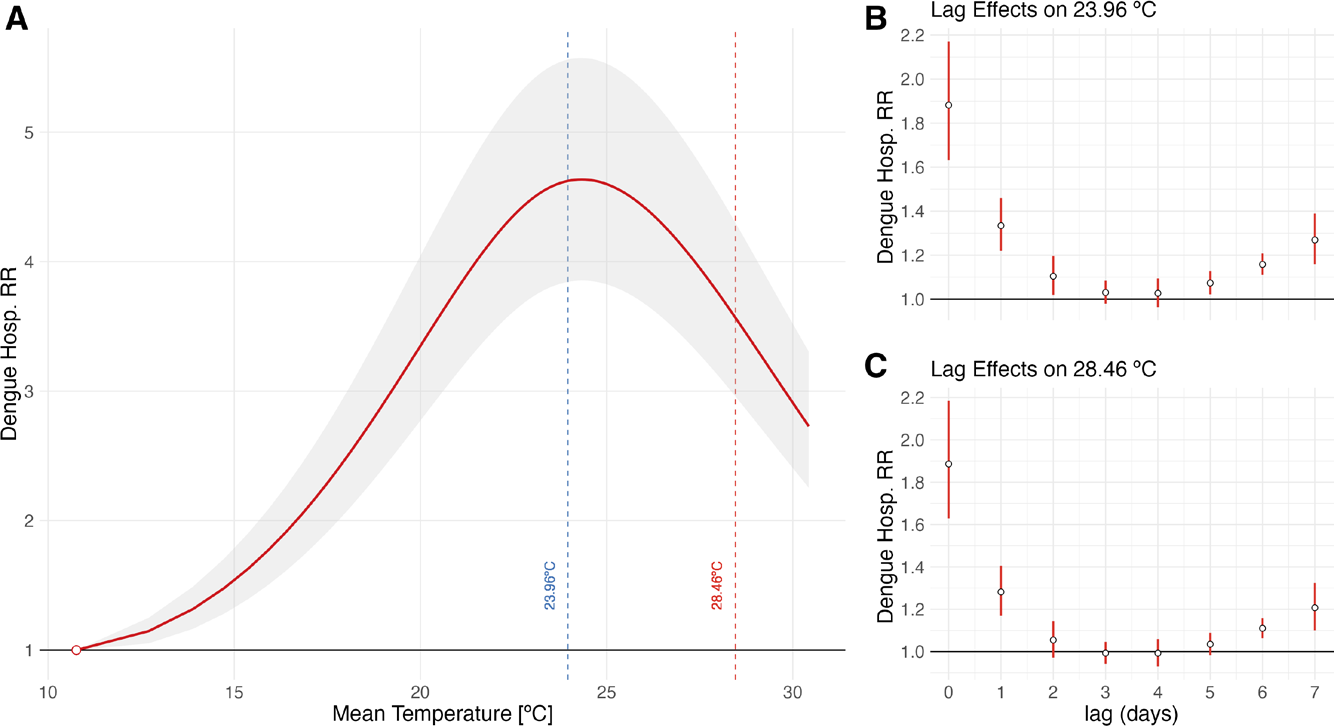
Dengue hospitalization relative risk by temperature in Brazil. A, The red line is the estimated relative risk of dengue hospitalization cumulated overall the lags considered as a function of temperature in degree Celsius compared with the minimal hospitalization temperature (MHT). The shaded gray area around the red line is the confidence interval of the estimated relative risk. The dashed blue line marks the 50th temperature percentile, at 23.96 ºC, and the dashed red line marks the 95th temperature percentile, at 28.68 ºC. B and C, The lag effects at the specific temperature percentiles, respectively, at 50th and 95th temperature, centered on the MHT.

The overall cumulative exposure–response curve for the temperature–dengue hospitalization association across Brazil is shown in Figure 2. There is a clear increase in the RR of hospitalization as temperature rises above the MHT, peaking at the 50th percentile (4.62; 95% CI: 3.84, 5.56) before decreasing at higher temperatures. The lag structure showed increased risk from lag 0 to lag 2.

**Figure 3.**
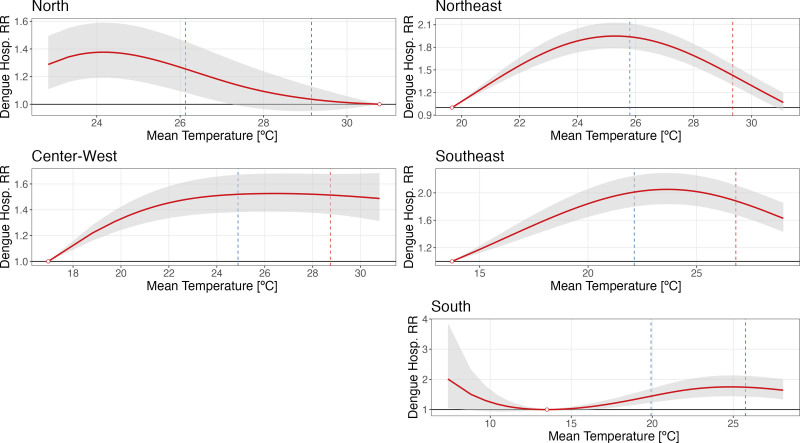
Dengue hospitalization relative risk by temperature in each Brazilian macroregion. The red line is the estimated relative risk of dengue hospitalization cumulated overall the lags considered as a function of temperature in degree Celsius compared with the minimal hospitalization temperature (MHT) for each macroregion. The shaded grey area around the red line is the confidence interval of the estimated relative risk. The dashed blue line marks the 50th temperature percentile and the dashed red line marks the 95th temperature percentile for each macroregion.

Figure 3 shows the overall cumulative exposure–response curves for each macroregion. The shape of the association is consistent with the national pattern in the Northeast, Center-West, Southeast, and South regions. However, the South region exhibits an unclear pattern below the MHT, and there is great uncertainty about the estimates in the North region. Lag-specific associations for each macroregion are shown in Figures A2–A6; http://links.lww.com/EE/A318. A summary of the RR and MHT for Brazil and each macroregion is presented in Table 2. The overall cumulative exposure–response curves at the state level are shown in Figure A7; http://links.lww.com/EE/A318.

**Table 2.** Dengue hospitalization relative risk (RR) over Brazil and each macroregion in the period of 2010–2019

Unit	Minimum hospitalisation temperature (ºC)	RR (95% CI) at 50th percentile (23.96 ºC)	RR (95% CI) at 95th percentile (28.68 ºC)
Brazil	10.8	4.62 (3.84, 5.56)	3.47 (2.88, 4.19)
North	30.8	1.26 (1.08, 1.45)	1.03 (0.95, 1.11)
Northeast	19.7	1.94 (1.78, 2.12)	1.39 (1.26, 1.53)
Center-West	17.0	1.52 (1.38, 1.67)	1.51 (1.36, 1.68)
Southeast	13.7	2.01 (1.80, 2.25)	1.86 (1.66, 2.09)
South	13.5	1.44 (1.23, 1.70)	1.74 (1.43, 2.11)

The relative risks were estimated by comparing the temperature at the 50th and 95th percentiles to the minimum hospitalisation temperature. These were estimated using a Case Times Series design, applying a cross-basis parametrized with a natural spline with two knots at the exposure domain and a natural spline with two knots equally spaced in the log scale in the 7-day lag structure. The estimates for Brazil were obtained on a single-stage model for the whole country, as it was estimated for each macroregion.

The overall cumulative exposure–response curves were similar by age and sex, with a greater magnitude of the association for females and those aged ≥65 years (Table 3).

**Table 3.** Dengue hospitalization relative risk (RR) overall and stratified by age and sex in Brazil in the period of 2010–2019

Subgroup	RR (95% CI) at 50th percentile (23.96 ºC)	RR (95% CI) at 95th percentile (28.68 ºC)
Overall	4.62 (3.84, 5.56)	3.47 (2.88, 4.19)
Age		
<65	4.39 (3.62, 5.32)	3.34 (2.74, 4.05)
≥65	7.20 (4.08, 12.69)	5.06 (2.85, 9.00)
Sex		
Males	3.94 (3.10, 5.00)	2.92 (2.29, 3.72)
Females	5.65 (4.34, 7.36)	4.30 (3.29, 5.61)

The relative risks were estimated by comparing the temperature at the 50th and 95th percentiles to the minimum hospitalisation temperature for Brazil (10.8 ºC). These were estimated using a Case Times Series design, applying a cross-basis parametrized with a natural spline with two knots at the exposure domain and a natural spline with two knots equally spaced in the log scale in the 7-day lag structure. The estimates were obtained on a single-stage model for the whole country, as it was estimated for each subgroup.

### Sensitivity analyses

**Figure 4.**
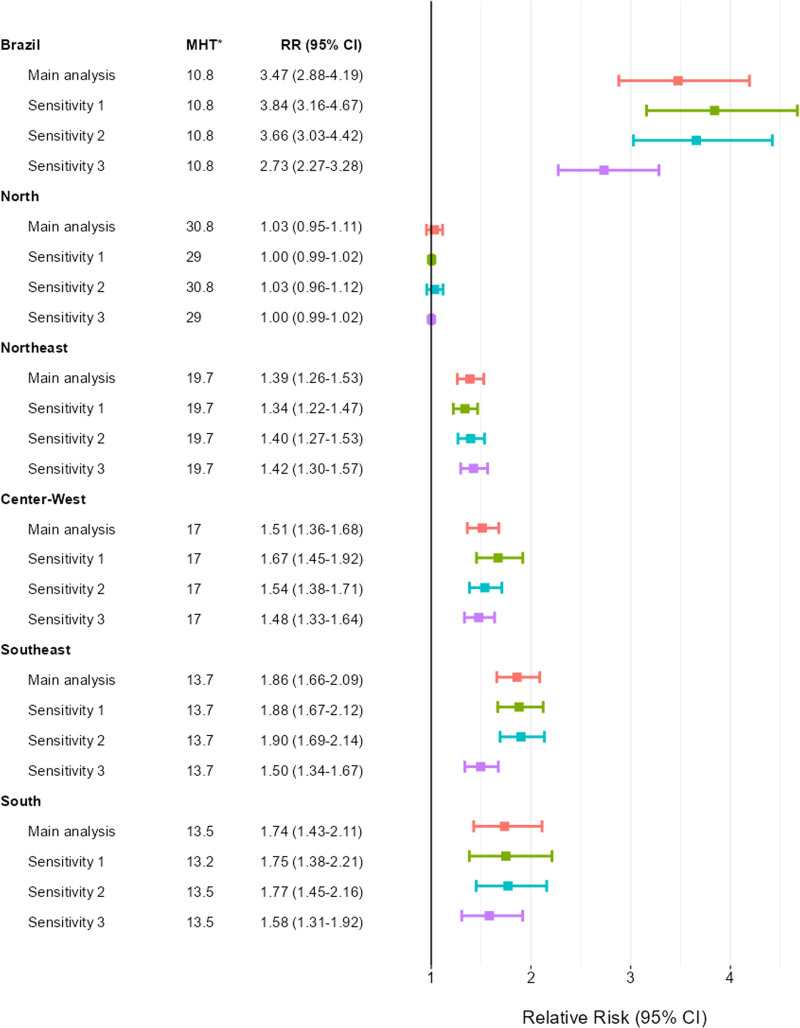
Dengue hospitalization relative risk by Brazil and each macroregion: main and sensitivity analyses at 95th percentile of temperature. The relative risks were estimated by comparing the temperature at the 95th percentile to the minimum hospitalization temperature. These were estimated using a Case Times Series design, applying a cross-basis parametrized with a natural spline with two knots at the exposure domain and a natural spline with two knots equally spaced in the log scale in the 7-day lag structure (main analysis). In the Sensitivity 1 models, we used three knots at the exposure domain; in the Sensitivity 2 models, we used three knots equally spaced in the log scale for the lag structure; in the Sensitivity 3 models, we used the same parametrization as the main analysis but adjusted for the 7-day moving average of the number of dengue cases. The estimates for Brazil were obtained on a single-stage model for the whole country, as it was estimated for each macroregion. The same figure is drawn for the 50th of temperature in the Appendix (Figure A8). *MHT on degree Celsius.

Overall, the sensitivity analyses yield results comparable to the main analysis for Brazil (Figure 4 and Figure A8; http://links.lww.com/EE/A318). After adjusting for the number of dengue cases (third sensitivity analysis), the RR at the 95th percentile for Brazil decreased from 3.47 (95% CI: 2.88, 4.19) to 2.73 (95% CI: 2.27, 3.28). The exposure–response function and lag structure for the sensitivity analyses are shown in Figures A9–A11; http://links.lww.com/EE/A318. Similar patterns were observed across all macroregions in the sensitivity analyses.

## Discussion

Our main finding is that the RR of hospitalization due to dengue infection increases during periods of high temperature, in the short term, with the greater RR observed between lags 0 and 2. This pattern was consistent across all macroregions of Brazil, except for the North.

The association between temperature and hospitalization due to vector-borne diseases has been scarcely explored in the literature. To our knowledge, this is the first study to quantify the association between temperature and dengue-related hospitalizations on a nationwide scale. Interestingly, our study did not observe a U-, V-, J-shaped curve seen in studies of ambient temperature and all-cause mortality and all-cause hospitalization.^17,18,31–34^ Indeed, the exposure–response function between ambient temperature and all-cause mortality usually is nonlinear, starting with high RR at low temperatures and decreasing as the temperature gets warmer values to a minimum, the minimum mortality temperature or the comfort temperature. Together with the symmetric phenomenon at high temperatures, the final exposure–response function is a U-shaped association curve. Similar to the minimum mortality temperature, we can statistically derive the MHT. When evaluating the MHT for dengue hospitalizations, we observed an increasing risk as the temperature gets warmer, with a maximum value to the RR and a decrease right after this maximum (increasing-saturating shape). A similar shape for MHT has been reported for studies on cause-specific hospitalizations, such as for cardiovascular hospitalizations in Japan and China, among others.^35–38^ In a nationwide study in Japan from 2011 to 2018,^35^ Pan et al conducted a time-stratified case-crossover analysis followed by a meta-analysis (two-stage design), observing increased risk for cardiovascular hospitalization only as the temperature gets colder, with their MHT at the maximum temperatures. In contrast, the other two studies conducted in China observed a similar pattern as our study,^37,38^ with an increased risk of cardiovascular hospitalizations as the temperature increases, with the MHT at the minimum temperatures.

It is worth mentioning that some specific features of dengue may explain the fact of not having a U-shaped association curve for the RR. First, dengue incidence, regardless of severity, is much lower during cold and dry months, reducing the power to detect any effect of low temperatures on hospitalizations and limiting the comparison between regions. This phenomenon is mainly related to the mosquito life cycle and the lower activity of mosquitoes at low temperatures. This highlights a common challenge in studying the relationship between climate variables and the severity of vector-borne diseases.^39^ Second, the progression of an infection of dengue to severe dengue is closely associated with factors like dehydration and capillary leak syndrome, which are augmented by high temperatures.

The observed association in terms of lag effects may be driven by multiple mechanisms. The immediate increase in RR at lag 0 is consistent with findings from other studies on high temperature and heatwaves and hospitalizations and mortality.^18,19,40–43^ This immediate effect likely reflects the worsening of clinical conditions due to high temperatures. The sharp increase at lag 0 strongly suggests that our results reflect a direct effect of ambient temperature on hospitalization risk, rather than an indirect effect through increased mosquito activity, which typically operates on a longer timescale (months).^4^ To further evaluate this issue, we conducted a sensitivity analysis (sensitivity analysis 3) adjusting for the 7-day rolling average of total dengue cases (mild and severe), aiming to capture the potential effect of temperature on dengue incidence in the short term. We observed a reduction in the point-estimate of the association, but the clear shape of increased RR of hospitalization still remains. Our findings contribute to the growing body of literature on the impact of temperature on dengue, showing that high temperatures have both short- and long-term effects on the burden of the disease.

Our study has several strengths. We analyzed a comprehensive nationwide database, providing a 10-year time series of dengue hospitalizations in a low- and middle-income country that experiences a wide range of temperatures. We also utilized temperature data from ERA5-Land reanalysis products,^41^ which may enhance the generalizability of our findings to other low- and middle-income countries, especially those where dengue has been expanding in recent years. Additionally, we employed the DLNM approach, which accounts for both dose–response and lag–response structures, as well as the correlation in daily temperature data.^26,27^

Our study has limitations to be mentioned. First, we did not account for other factors that could modify the effects of high temperatures, such as green spaces, urbanization, and relative humidity.^44^ Second, although we analyzed nearly 580,000 events across 5565 municipalities over a decade, the precision of our estimates could still be limited. Care is advised when extrapolating or interpreting our results to any other infectious disease or vector-borne disease. Third, we evaluated temperature–dengue hospitalization associations at two administrative levels, the whole country and macroregions. While this approach allows for comparisons among different regions but with similar climate and dengue incidence, it may limit the generalizability of our results to specific municipalities within each state or to borderline areas. Finally, there is potential for exposure misclassification when using ERA5-Land reanalysis data, particularly in the North region. The North region concentrated the lowest *R*^2^ values when comparing the ERA5-Land estimates with the ground station measurements (see ERA5-Land Validation; http://links.lww.com/EE/A318). Nonetheless, the use of daily average temperature from ERA5-Land reanalysis has been validated in several epidemiological studies,^45,46^ with minimal differences observed in measures of effect estimates when comparing those obtained using ERA5-Land to weather station data.^41,47^ Therefore, we do not expect meaningful impacts on our results due to the potential exposure misclassification. Additionally, the average temperature range at the North region is limited to high temperatures, with 95% of the temperatures in the range of 23.91–29.32 ºC, which might have limited our power in the analysis there because of limited contrast in the exposure range.

In conclusion, we observed an association between high temperature and increased RR of dengue hospitalization in Brazil, driven primarily by the immediate effects of heat. This association varied across the five Brazilian macroregions, with an unclear pattern in the North. This study fills a gap in the literature by demonstrating a short-term effect of temperature on dengue severity controlling for incidence and disease burden, complementing the well-documented long-term relationship between temperature and the disease incidence.

## Conflicts of interest statement

The authors declare that they have no conflicts of interest with regard to the content of this report.

## Supplementary Material


