# Supplementary material for: Ambient temperature and dengue hospitalization in Brazil: A 10-year period case time series analysis
Source: Environ Epidemiol. 2024 Dec 30;9(1):e360. doi: 10.1097/EE9.0000000000000360 (PMC11688019; doi:10.1097/EE9.0000000000000360)
Supplement: Supplementary file 1 [file ee9-9-e360-s001.pdf]

Supplementary Material

Ambient Temperature and Dengue Hospitalisation in Brazil: A 10-year period case time series analysis

Lopes R, et al.

Table of Contents

Model description and Case time series design 3

ICD-10 codes definition 4

ERA5-Land validation 5

Table A1. Cross-basis parameter selection specification 7

Table A2. Descriptive distribution of 2m height Mean Temperature 8

Figure A1. Time series of Dengue hospitalisation by macro administrative region of Brazil. Colour by year, the data covers a period of 10 years, from the whole epidemiological year of 2010 to the whole epidemiological year of 2019. 9

Table A3. Brazilian population demographic information from the national Census-2010 10

Figure A2. A) Cumulative relative risk over all lags for a Dengue hospitalisation compared to the MHT at the North Region. B) Lag effect of the RR on the 50th percentile of temperature. C) Lag effect of the RR on the 95th percentile of temperature (Main analysis) 11

Figure A3. A) Cumulative relative risk over all lags for a Dengue hospitalisation compared to the MHT at the Northeast Region. B) Lag effect of the RR on the 50th percentile of temperature. C) Lag effect of the RR on the 95th percentile of temperature (Main analysis). 12

Figure A4. A) Cumulative relative risk over all lags for a Dengue hospitalisation compared to the MHT at the Center-West Region. B) Lag effect of the RR on the 50th percentile of temperature. C) Lag effect of the RR on the 95th percentile of temperature (Main analysis). 13

Figure A5. A) Cumulative relative risk over all lags for a Dengue hospitalisation compared to the MHT at the Southeast Region. B) Lag effect of the RR on the 50th percentile of temperature. C) Lag effect of the RR on the 95th percentile of temperature (Main analysis). 14

Figure A6. A) Cumulative relative risk over all lags for a Dengue hospitalisation compared to the MHT at the South Region. B) Lag effect of the RR on the 50th percentile of temperature. C) Lag effect of the RR on the 95th percentile of temperature (Main analysis) 15

Figure A7. Cumulative relative risk over all the lags compared to the MHT on each state level, for the whole period of analysis, 2010 to 2019 (First stage, Main Analysis) 16

Figure A8. Dengue hospitalization relative risk by Brazil and each macro-region: main and sensitivity analyses forest plot at 50th percentile of temperature 17

Figure A9. A) Cumulative relative risk over all lags for a Dengue hospitalisation compared to the MHT in Brazil. B) Lag effect of the RR on the 50th percentile of temperature. C) Lag effect of the RR on the 95th percentile of temperature (Sensitivity Analysis 1) 18

Figure A10. A) Cumulative relative risk over all lags for a Dengue hospitalization compared to the MHT in Brazil. B) Lag effect of the RR on the 50th percentile of temperature. C) Lag effect of the RR on the 95th percentile of temperature (Sensitivity Analysis 2) 19

Figure A11. A) Cumulative relative risk overall lags for a Dengue hospitalization compared to the MHT in Brazil. B) Lag effect of the RR on the 50th percentile of temperature. C) Lag effect of the RR on the 95th percentile of temperature (Sensitivity Analysis 3) 20

Model description and Case time series design

We employed a case time series design to model the association between temperature and hospitalization by dengue infection. We ran the following model for the whole country and for each macro-region. We suppose the assumption of the Dengue hospitalizations counts being given by a *quasi-Poisson* distribution, where the mean of the counts at *i-th* municipality at day *t* it is the $\lambda_{i,t}$and ψ is the parameter to account for over dispersion, the whole model it is then:

$$Y_{i,t} = quasi - Poisson(\lambda_{i,t}, \psi),$$

$$g[E(Y_{i,t})] = log[E(Y_{i,t})] = \xi_{i(k)} + f(x_{i,t}, l) + \sum_{j=1}^{J} s_j(t)$$

Where $Y_{i,t}$ is the daily count of hospitalizations on the *i-th* municipality, $\xi_{i(k)}$ the baseline risks varying across *i-th* municipalities, stratified further by defining different intercepts for each time stratum k: day of the week (*dow*) and month, defining *dow*-month-municipality strata term conditioned out. $f(x_{i,t}, l)$ which is the bi-dimensional exposure-lag-response function given by a distributed lag non-linear model for mean temperature by each day of delay, until 7 days of lags. The cross-basis is parametrized with a natural spline, with 2 knots equally spaced on the exposure-response structure and 3 knots equally spaced on the log-transformed scale for the lag-response structure. The last term is the long-term trend model choice for temperature trend along the whole period, a natural spline with 7 degrees of freedom by each year on the whole period.

ICD-10 codes definition

We gathered only the hospitalization with the following ICD-10 codes: 'A90', 'A91', 'A97', 'A970', 'A971', 'A972', 'A979'. Below we give their descriptive definitions. These codes availability changed during different versions of ICD-10.

‘A90’ - Dengue fever [classical dengue]
‘A91’ - Dengue hemorrhagic fever
‘A97’ – Dengue

Dengue is a viral disease transmitted by the bite of a mosquito infected by dengue viruses. It is one disease entity with different clinical presentations and often with unpredictable clinical evolution and outcome. Most patients recover following a self-limiting non-severe clinical course like nausea, vomiting, rash, aches and pains, but a small proportion progress to severe disease, mostly characterized by plasma leakage with or without haemorrhage, although severe haemorrhages or severe organ impairment can occur, with or without dengue shock.

‘A970’ - Dengue without warning signs

Dengue haemorrhagic fever grades 1 and 2. Dengue haemorrhagic fever without warning signs

‘A971’ - Dengue with warning signs

Clinical warning signs are: abdominal pain or tenderness, mucosal bleeding, lethargy and /or restlessness, rapid decrease in platelet count, increase in hematocrit. Other signs can include: persistent vomiting, visible fluid accumulation, liver enlargement more than 2 cm.

Dengue haemorrhagic fever with warning signs

‘A972’ - Severe Dengue

Clinical signs include: 1. Severe plasma leakage leading to shock (Dengue shock syndrome - DSS) and/or fluid accumulation with respiratory distress; 2. Severe bleeding as evaluated by clinician; 3. Severe organ involvement: Liver AST or ALT >=1000, CNS: impaired consciousness (encephalitis), involvement of other organs, as myocarditis or nephritis

Severe dengue fever
Severe dengue haemorrhagic fever

‘A979’ - Dengue, unspecified

Dengue fever [DF] NOS

ERA5-Land validation

We performed a validation comparing the daily average of temperature from monitoring stations at the municipal level with the ERA5-Land estimates. We used data from 389 stations (269 automatic and 120 manual) from the National Institute of Meteorology (INMET), with at least 90% of days with complete data during the period. This validation has a total of 1,221,051 days evaluated, from 340 municipalities covering the 27 states and the 5 macro-regions of Brazil.

	**R-Pearson**	**R-square**	**RMSE**
Overall	0.94	0.90	1.54
Distribution across 340 municipalities			
Mean ± SD	0.89 ± 0.1	0.81 ± 0.1	1.02 ± 0.3
Median [p25-p75]	0.92 [0.86-0.95]	0.85 [0.74-0.90]	1.01 [0.85-1.17]

Spatial distribution of R-square for each of the 340 municipalities. The shade areas are the municipality areas. N stands for North (n=39), NE for Northeast (n=95), CW for Center-West (n=42), SE for Southeast (n=103) and S for South (n=61).

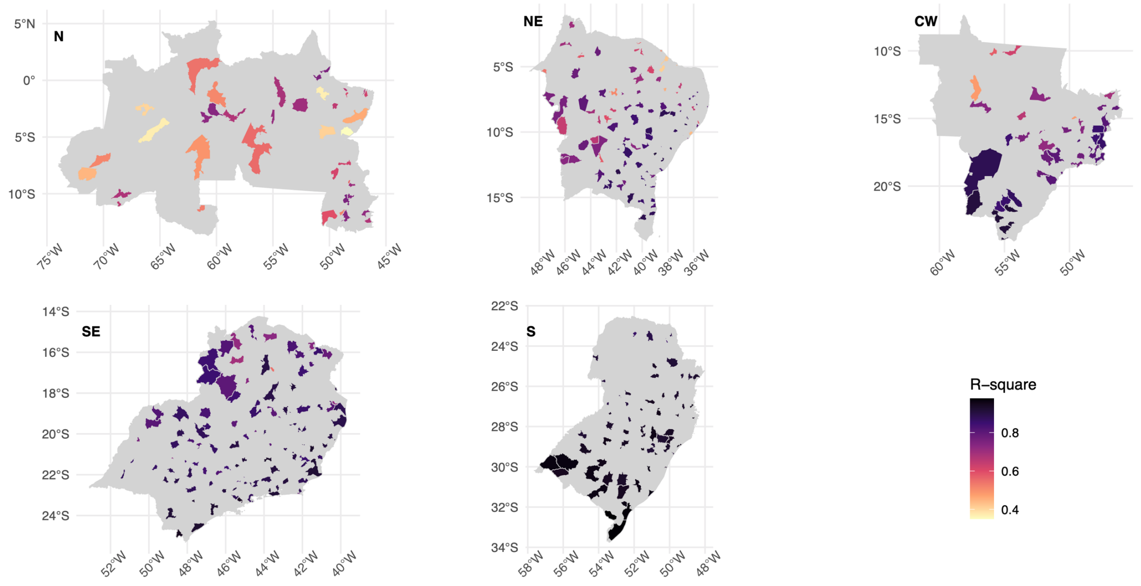

Spatial distribution of RMSE for each of the 340 municipalities. The shade areas are the municipality areas. N stands for North (n=39), NE for Northeast (n=95), CW for Center-West (n=42), SE for Southeast (n=103) and S for South (n=61).

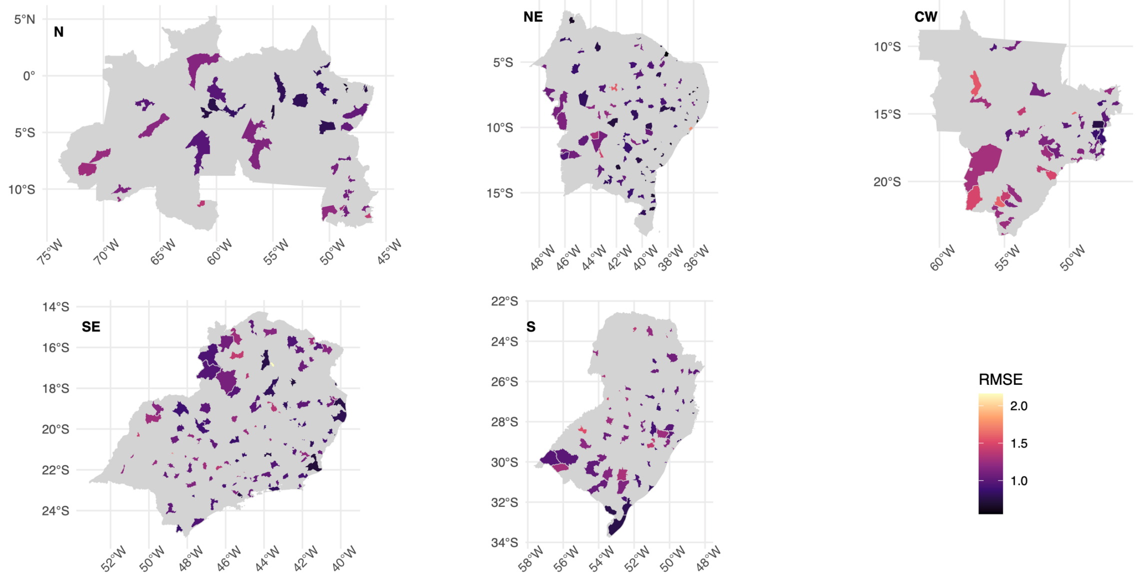

Table A1. Cross-basis parameter selection specification

Exposure-response function	Lag	qAIC
2 knots, ns (MAIN)	2 knots, equally spaced log scale (MAIN)	3025660.1
3 knots, ns	2 knots, equally spaced log scale (MAIN)	3025700.7
1 knot, ns	2 knots, equally spaced log scale (MAIN)	3026053.3
Linear	2 knots, equally spaced log scale (MAIN)	3028889.9

Table A2. Descriptive distribution of 2m height Mean Temperature

Brazil, Region, State	Minimum	5th	25th	50th	75th	95th	Maximum
Brazil	-0.09	15.39	20.92	23.96	26.08	28.68	34.8
North	13.99	23.91	25.15	26.13	27.39	29.32	34.32
AC	13.99	22.81	24.35	25.13	26.01	27.49	31.01
AM	17.98	24.18	25.09	25.81	26.68	28.39	33.06
AP	21.98	23.72	24.73	25.65	27.14	28.59	30.5
PA	21.7	24.24	25.38	26.31	27.53	28.99	32.59
RO	14.11	23.34	24.67	25.52	26.6	28.59	31.61
RR	20.04	22.88	24.29	25.33	26.67	28.82	32.24
TO	19.6	24.17	25.58	26.71	28.03	30.27	34.32
Northeast	14.04	21.49	24.16	25.8	27.24	29.54	34.39
AL	18.6	21.76	23.52	25.02	26.32	27.9	32.78
BA	14.04	19.99	22.31	24.08	25.73	27.8	33.99
CE	19.09	23.82	25.58	26.77	27.81	29.16	32.41
MA	21.75	24.53	25.79	26.84	28.09	29.89	33.29
PB	19.17	22.36	24.19	25.49	26.74	29.15	32.08
PE	17.59	20.77	22.95	24.35	25.76	27.84	32.71
PI	20.71	24.44	26.07	27.55	29.3	31.41	34.39
RN	20.18	24.02	25.56	26.6	27.66	29.29	31.86
SE	19.77	22.17	23.74	25.35	26.53	27.77	32.25
Center-West	6.09	20.68	23.34	24.88	26.41	28.96	34.3
DF	15.96	19.42	21.01	22.23	23.54	25.87	29.58
GO	11.3	20.99	22.97	24.4	26	28.76	34.18
MS	6.09	16.95	22.42	24.71	26.46	28.81	34.26
MT	10.6	22.55	24.46	25.61	26.89	29.31	34.3
Southeast	4.69	16.13	19.7	22.13	24.31	27.03	34.8
ES	10.78	17.22	20.39	22.49	24.66	26.82	31.46
MG	6.71	16.15	19.47	21.76	23.82	26.62	34.54
SP	4.69	15.88	19.96	22.66	24.83	27.45	34.8
RJ	9.78	16.75	19.79	22.12	24.45	27.2	32.25
South	-0.09	11.03	16.52	19.92	22.76	26.02	33.03
PR	0.67	13.25	18.19	21.27	23.78	26.78	32.42
RS	0.77	9.99	15.71	19.33	22.35	25.7	33.03
SC	-0.09	10.8	15.86	18.98	21.73	24.89	31.13

Figure A1. Time series of Dengue hospitalisation by macro administrative region of Brazil. Colour by year, the data covers a period of 10 years, from the whole epidemiological year of 2010 to the whole epidemiological year of 2019.

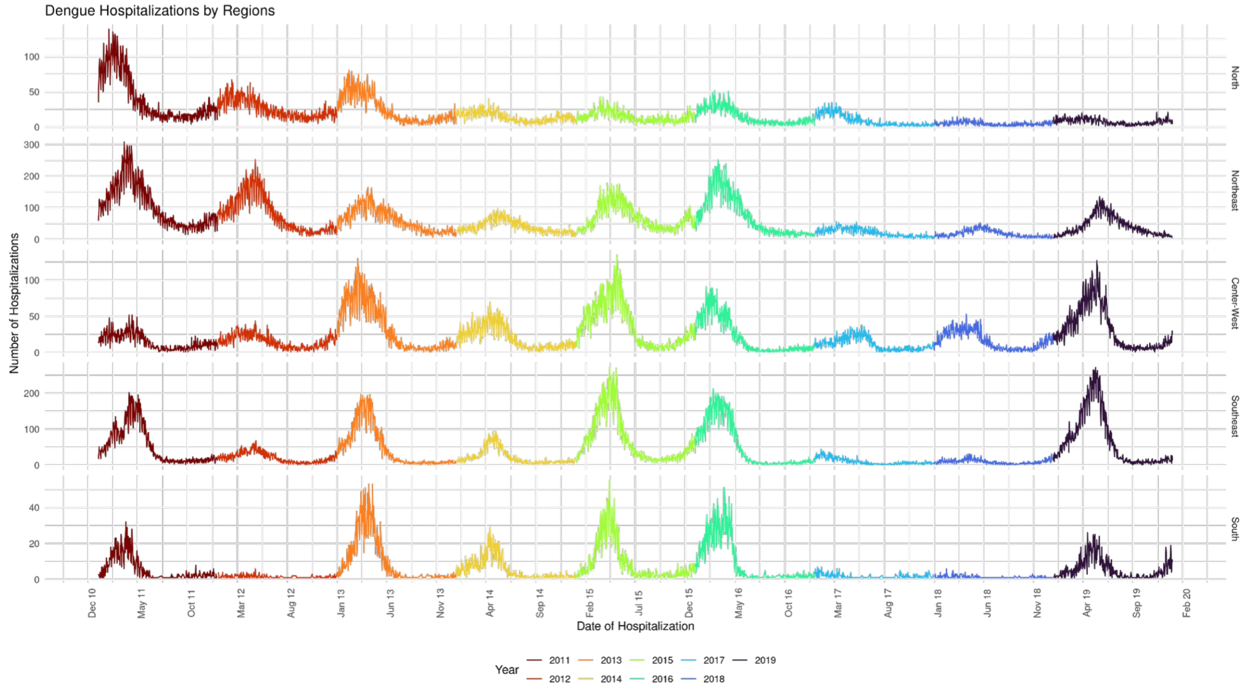

Table A3. Brazilian population demographic information from the national Census-2010

**Variables**	**Brazil**	**North**	**Northeast**	**Center-West**	**Southeast**	**South**
**Age, median, years**	29	24	27	28	31	31
**Age Categories, (%), years**						
0 to 1	1.4%	1.9%	1.6%	1.5%	1.3%	1.3%
1 to 9	13.7%	18.2%	15.1%	14.1%	12.2%	12.2%
10 to 17	12.7%	15.4%	13.9%	12.6%	11.6%	11.9%
18 to 39	37.4%	38.8%	37.8%	39.3%	37.0%	35.9%
40 to 59	23.3%	18.2%	20.6%	23.0%	25.2%	25.9%
60 to 79	9.8%	6.3%	9.1%	8.3%	10.8%	11.1%
80+	1.8%	1.0%	1.8%	1.2%	1.9%	1.8%
**Self-Reported Race, (%)**						
Black	7.5%	6.5%	9.4%	6.6%	7.8%	4.0%
Pardo	43.4%	67.2%	59.8%	49.5%	36.0%	16.7%
Indigenous	0.4%	1.9%	0.4%	0.9%	0.1%	0.3%
White	47.5%	23.2%	29.2%	41.5%	55.0%	78.3%
Asian	1.1%	1.1%	1.2%	1.5%	1.1%	0.7%
**Sex, (%)**						
Female	51.0%	49.5%	51.2%	50.3%	51.4%	50.9%
Male	49.0%	50.5%	48.8%	49.7%	48.6%	49.1%

Figure A2. A) Cumulative relative risk over all lags for a Dengue hospitalisation compared to the MHT at the North Region. B) Lag effect of the RR on the 50th percentile of temperature. C) Lag effect of the RR on the 95th percentile of temperature (Main analysis)

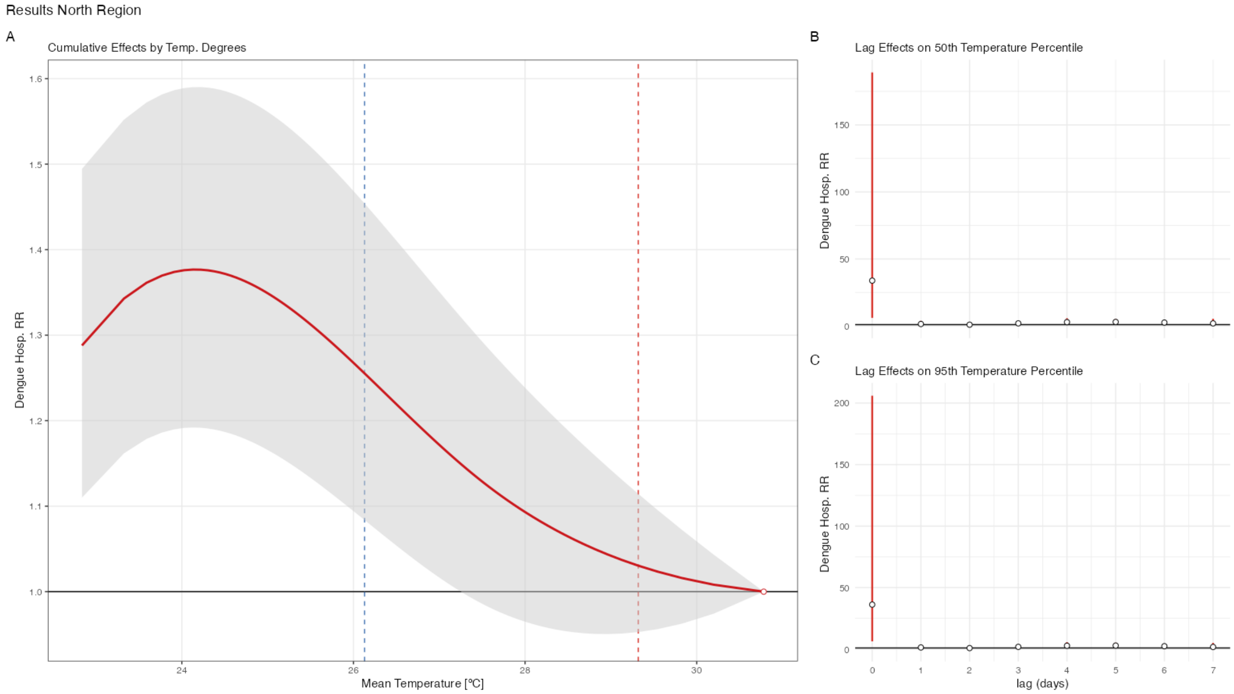

The grey shade (A) is 95% confidence interval, as the error bars (B and C), derived from the meta-analysis. The blue dashed line marks the 50th temperature percentile and the red dashed line marks the 95th temperature percentile.

Figure A3. A) Cumulative relative risk over all lags for a Dengue hospitalisation compared to the MHT at the Northeast Region. B) Lag effect of the RR on the 50th percentile of temperature. C) Lag effect of the RR on the 95th percentile of temperature (Main analysis)

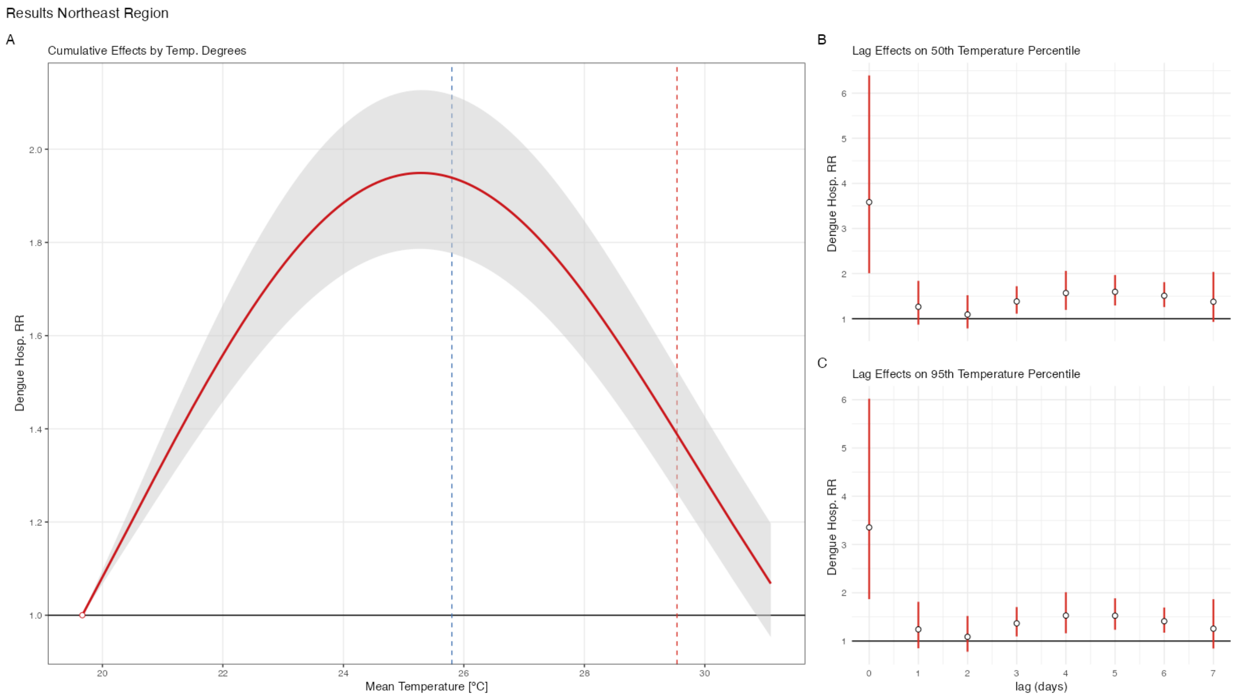

The grey shade (A) is 95% confidence interval, as the error bars (B and C), derived from the meta-analysis. The blue dashed line marks the 50th temperature percentile and the red dashed line marks the 95th temperature percentile.

Figure A4. A) Cumulative relative risk over all lags for a Dengue hospitalisation compared to the MHT at the Center-West Region. B) Lag effect of the RR on the 50th percentile of temperature. C) Lag effect of the RR on the 95th percentile of temperature (Main analysis)

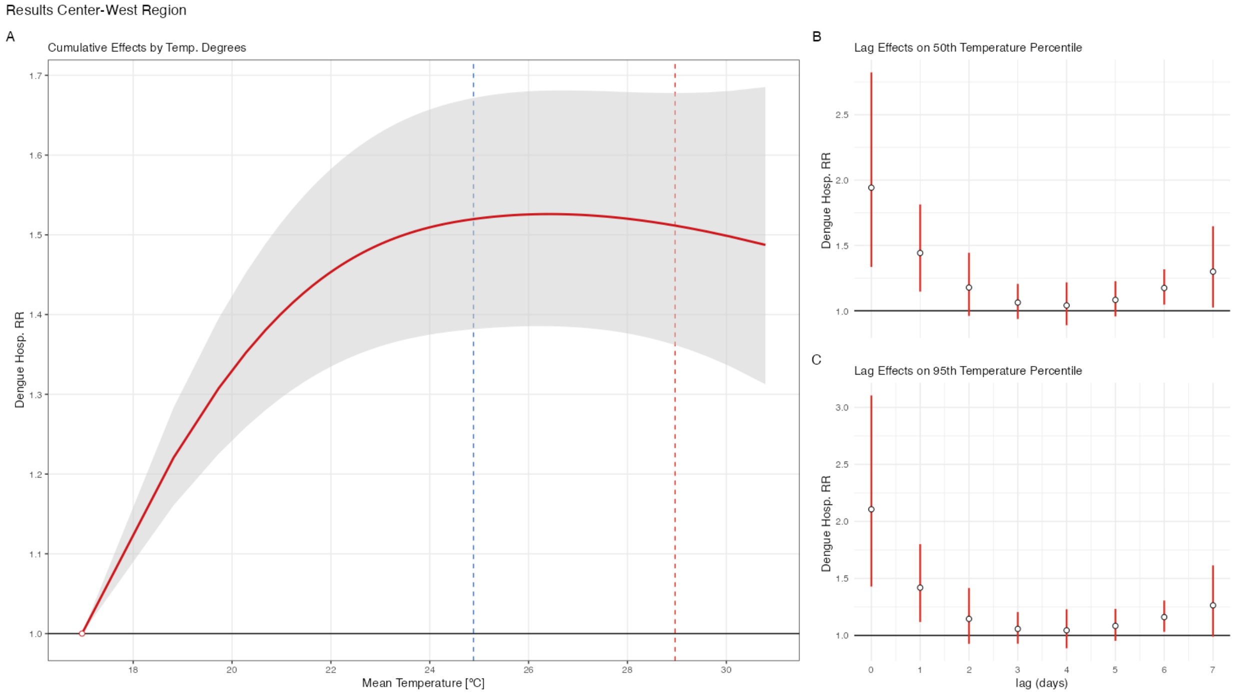

The grey shade (A) is 95% confidence interval, as the error bars (B and C), derived from the meta-analysis. The blue dashed line marks the 50th temperature percentile and the red dashed line marks the 95th temperature percentile.

Figure A5. A) Cumulative relative risk over all lags for a Dengue hospitalisation compared to the MHT at the Southeast Region. B) Lag effect of the RR on the 50th percentile of temperature. C) Lag effect of the RR on the 95th percentile of temperature (Main analysis)

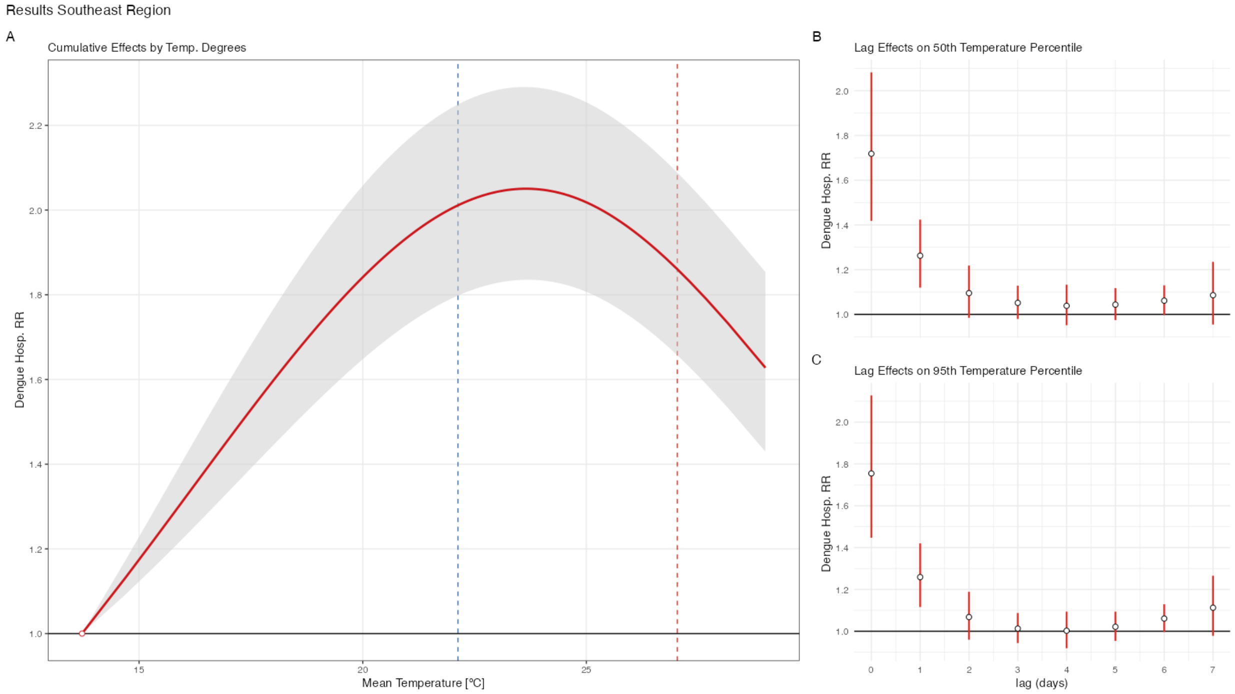

The grey shade (A) is 95% confidence interval, as the error bars (B and C), derived from the meta-analysis. The blue dashed line marks the 50th temperature percentile and the red dashed line marks the 95th temperature percentile.

Figure A6. A) Cumulative relative risk over all lags for a Dengue hospitalisation compared to the MHT at the South Region. B) Lag effect of the RR on the 50th percentile of temperature. C) Lag effect of the RR on the 95th percentile of temperature (Main analysis)

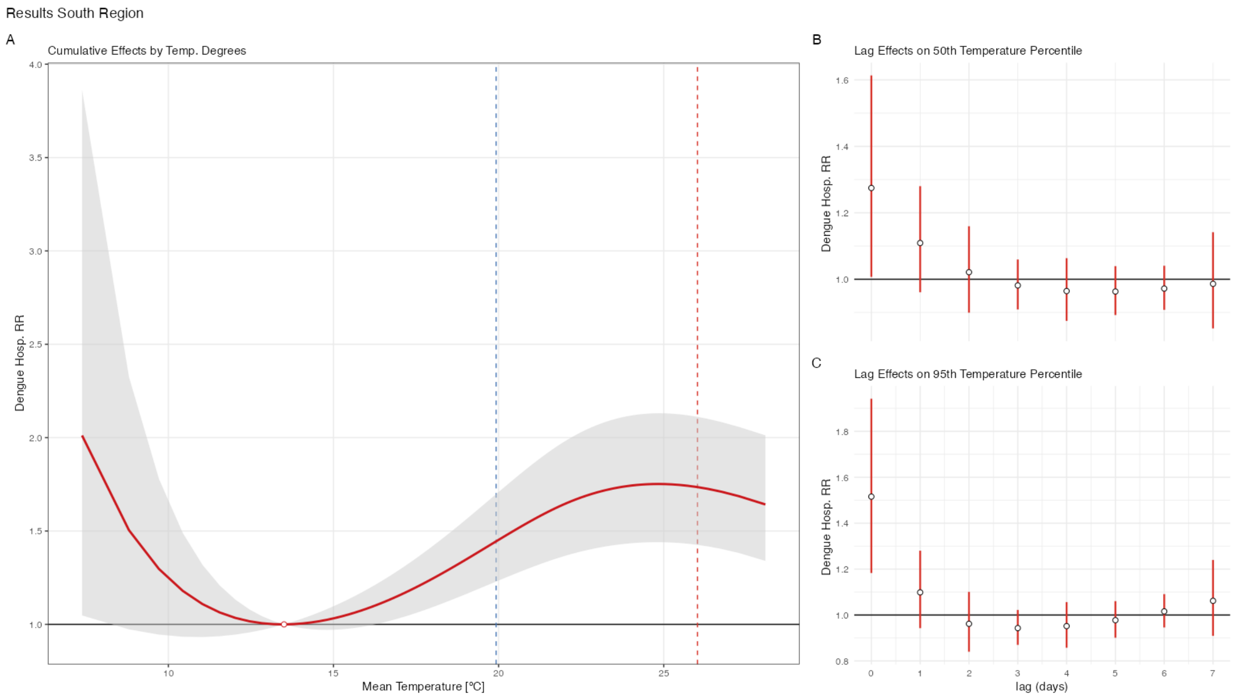

The grey shade (A) is 95% confidence interval, as the error bars (B and C), derived from the meta-analysis. The blue dashed line marks the 50th temperature percentile and the red dashed line marks the 95th temperature percentile.

Figure A7. Cumulative relative risk over all the lags compared to the MHT on each state level, for the whole period of analysis, 2010 to 2019 (First stage, Main Analysis)

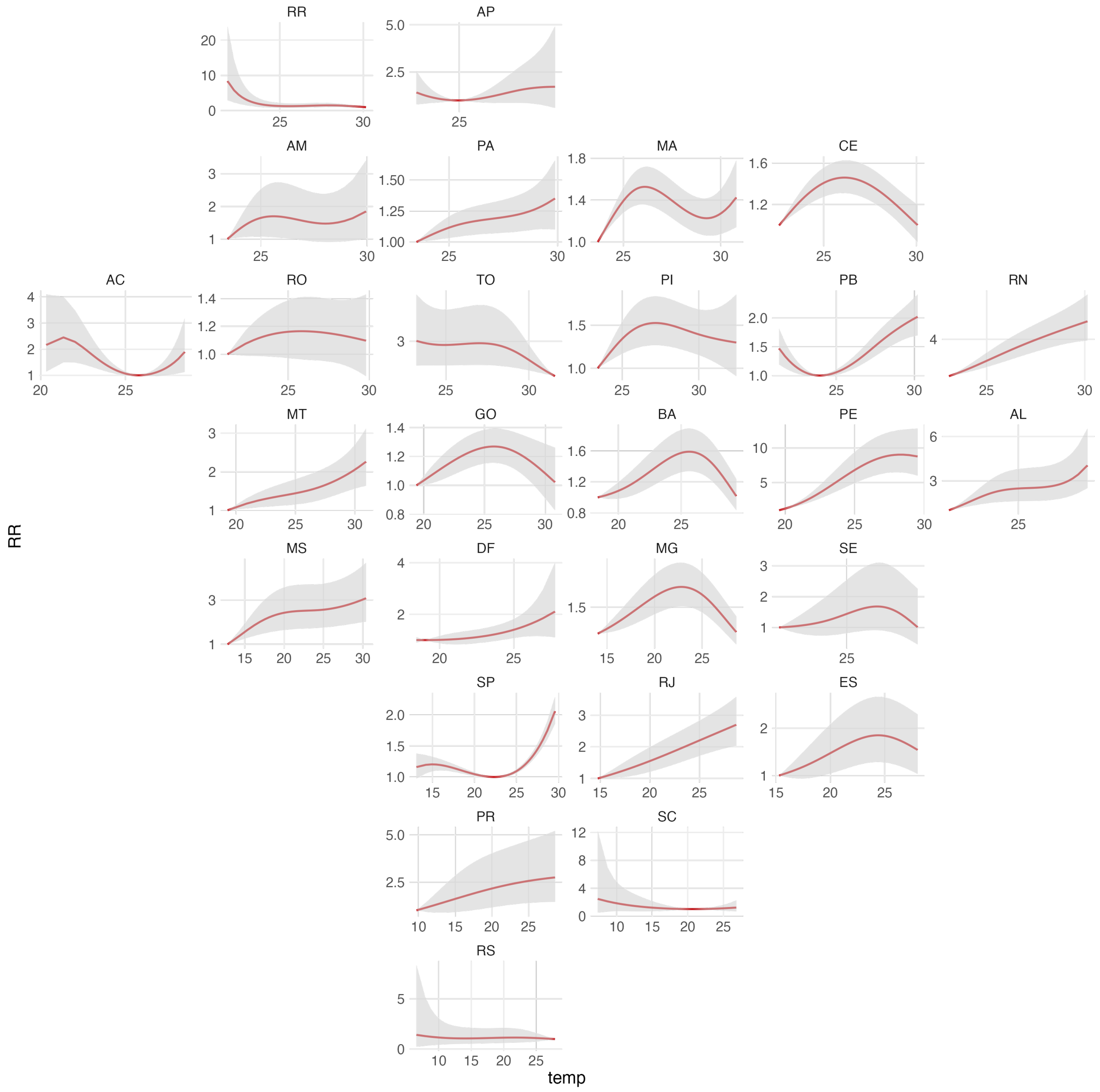

The curve is plotted in red lines and the 95% confidence interval generated from the fitted model is given by the grey shaded ribbon around it. The title of each subplot is the abbreviations for the name of each state.

Figure A8. Dengue hospitalization relative risk by Brazil and each macro-region: main and sensitivity analyses forest plot at 50th percentile of temperature

	MHT*	RR (95% CI)
**Brazil**		
Main analysis	10.8	4.62 (3.84-5.56)
Sensitivity 1	10.8	5.06 (4.16-6.14)
Sensitivity 2	10.8	4.87 (4.04-5.87)
Sensitivity 3	10.8	3.46 (2.89-4.15)
**North**		
Main analysis	30.8	1.26 (1.08-1.45)
Sensitivity 1	29	1.27 (1.19-1.35)
Sensitivity 2	30.8	1.26 (1.09-1.46)
Sensitivity 3	29	1.17 (1.10-1.24)
**Northeast**		
Main analysis	19.7	1.94 (1.78-2.12)
Sensitivity 1	19.7	1.88 (1.72-2.04)
Sensitivity 2	19.7	1.95 (1.78-2.13)
Sensitivity 3	19.7	1.95 (1.79-2.13)
**Center-West**		
Main analysis	17	1.52 (1.38-1.67)
Sensitivity 1	17	1.67 (1.47-1.89)
Sensitivity 2	17	1.55 (1.40-1.70)
Sensitivity 3	17	1.43 (1.30-1.57)
**Southeast**		
Main analysis	13.7	2.01 (1.80-2.25)
Sensitivity 1	13.7	2.03 (1.81-2.28)
Sensitivity 2	13.7	2.05 (1.84-2.30)
Sensitivity 3	13.7	1.55 (1.39-1.72)
**South**		
Main analysis	13.5	1.44 (1.23-1.70)
Sensitivity 1	13.2	1.43 (1.14-1.80)
Sensitivity 2	13.5	1.46 (1.24-1.72)
Sensitivity 3	13.5	1.36 (1.16-1.59)

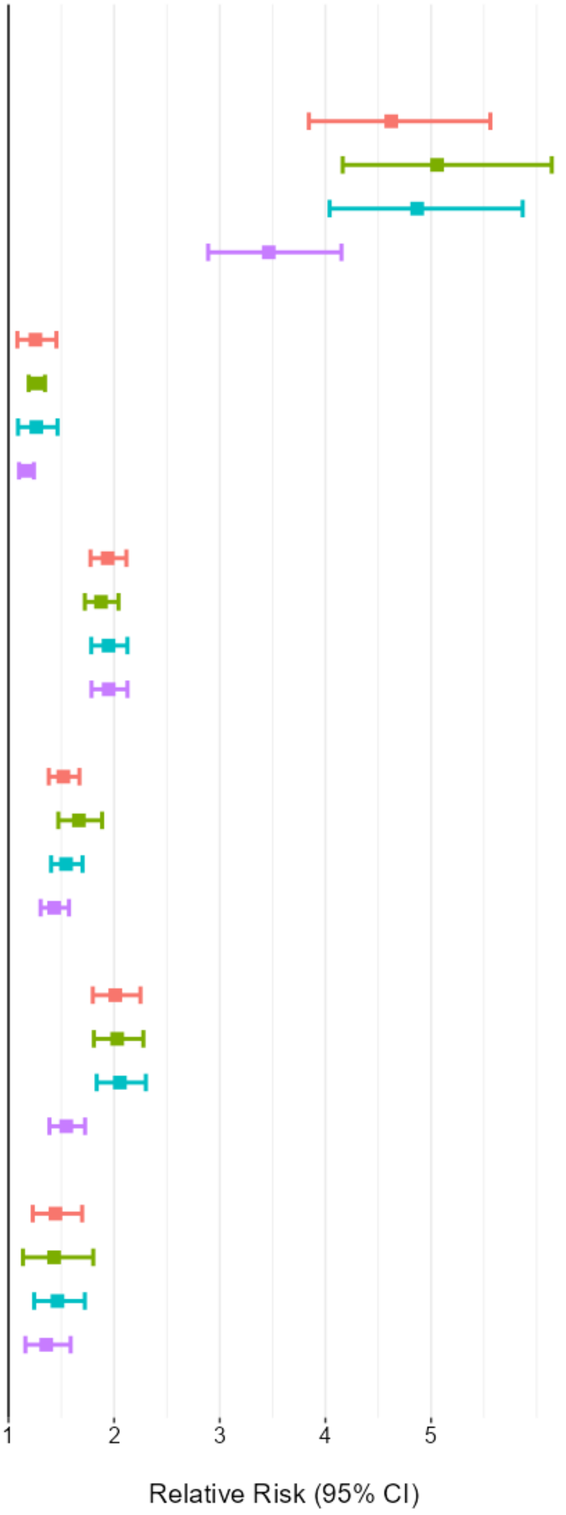

*MHT: Minimum Hospitalisation Temperature, on Celsius degree

Figure A9. A) Cumulative relative risk over all lags for a Dengue hospitalisation compared to the MHT in Brazil. B) Lag effect of the RR on the 50th percentile of temperature. C) Lag effect of the RR on the 95th percentile of temperature (Sensitivity Analysis 1)

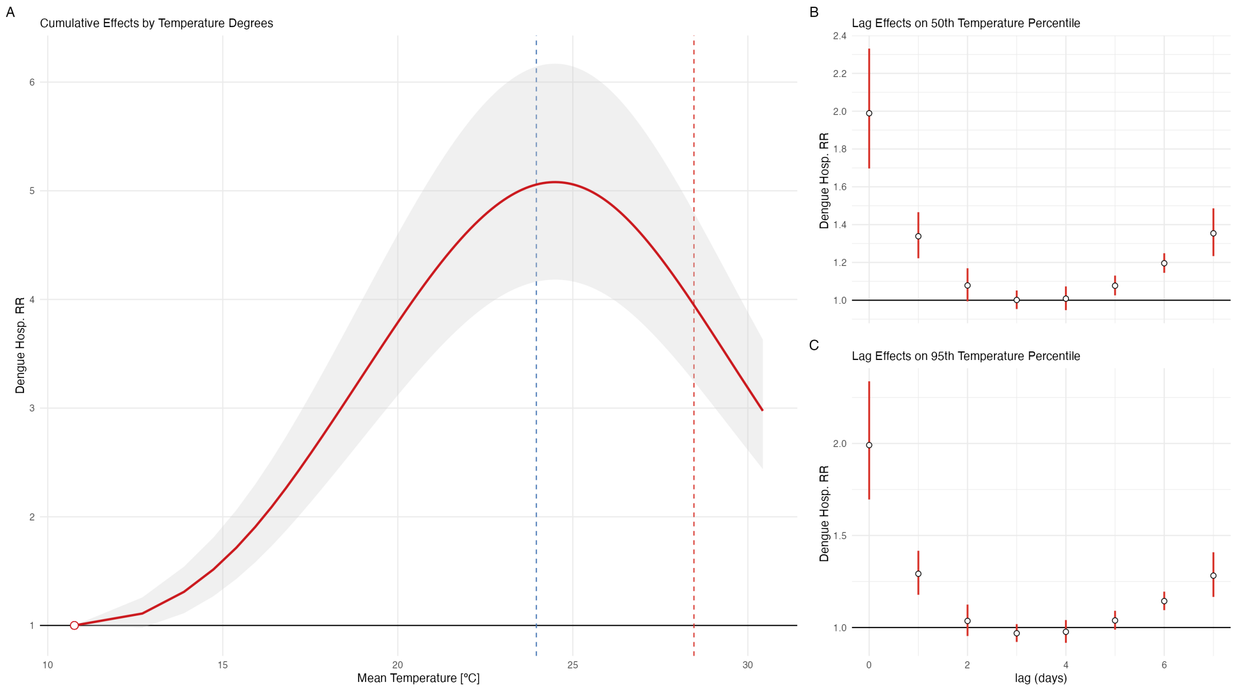

Sensitivity Analysis 1: parametrization of dose-response: 3 knots equally spaced; lag-response: 3 knots equally spaced at the log-scale. Vertical traced lines mark the 50th (Blue) and 95th (Red) percentile of the temperature distribution. The grey shade (A) is 95% confidence interval, as the error bars (B and C).

Figure A10. A) Cumulative relative risk over all lags for a Dengue hospitalization compared to the MHT in Brazil. B) Lag effect of the RR on the 50th percentile of temperature. C) Lag effect of the RR on the 95th percentile of temperature (Sensitivity Analysis 2)

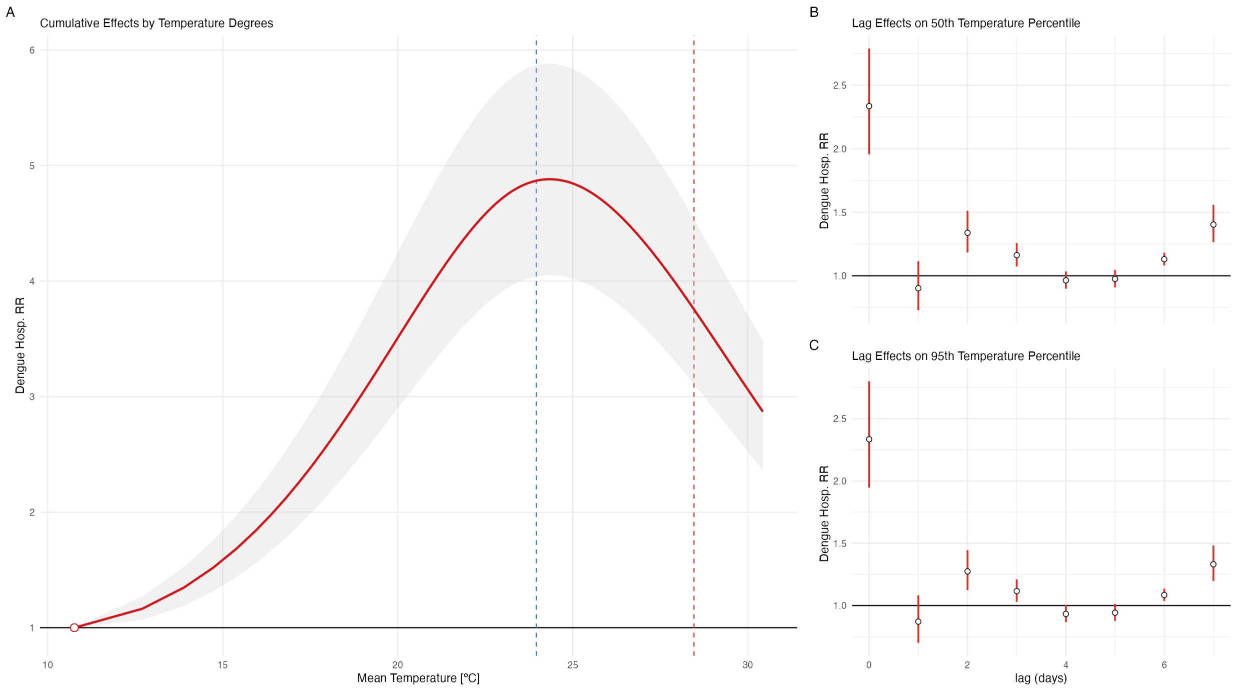

Sensitivity Analysis 2: parametrization of dose-response: 2 knots equally spaced; lag-response: 3 knots equally spaced at the log-scale. Vertical traced lines mark the 50th (Blue) and 95th (Red) percentile of the temperature distribution. The grey shade (A) is 95% confidence interval, as the error bars (B and C).

Figure A11. A) Cumulative relative risk overall lags for a Dengue hospitalization compared to the MHT in Brazil. B) Lag effect of the RR on the 50th percentile of temperature. C) Lag effect of the RR on the 95th percentile of temperature (Sensitivity Analysis 3)

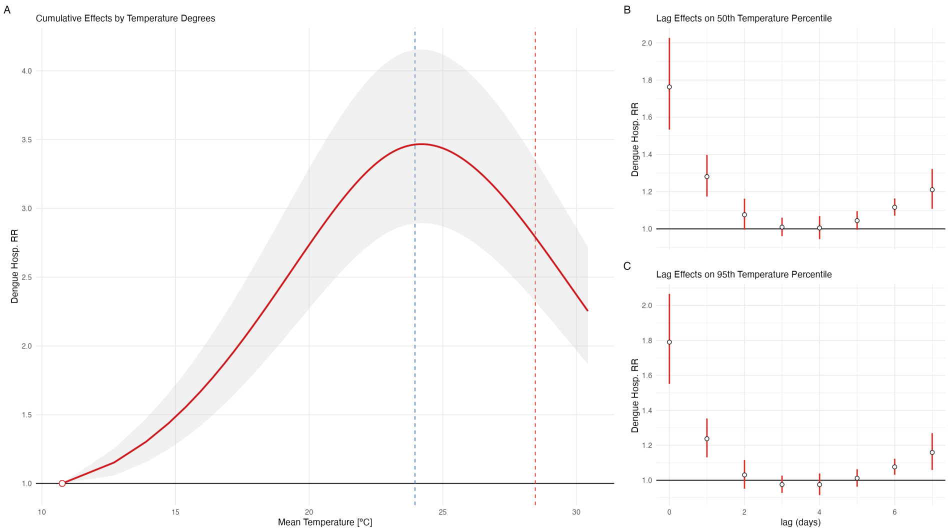

Sensitivity Analysis 3: parametrization of dose-response: 2 knots equally spaced; lag-response: lag-response: 3 knots equally spaced at the log-scale; covariate: 7-day moving average of confirmed dengue cases. Vertical traced lines mark the 50th (Blue) and 95th (Red) percentile of the temperature distribution. The grey shade (A) is 95% confidence interval, as the error bars (B and C).
